# Non-use of health care service among empty-nest elderly in Shandong, China: a cross-sectional study

**DOI:** 10.1186/s12913-015-0974-1

**Published:** 2015-07-29

**Authors:** Chengchao Zhou, Chunmei Ji, Jie Chu, Alexis Medina, Cuicui Li, Shan Jiang, Wengui Zheng, Jing Liu, Scott Rozelle

**Affiliations:** School of Public Health, Shandong University, Jinan, 250012 China; Licheng Centre for Maternal and Child Healthcare, Jinan, 250001 China; Shandong Centre for Disease Control and Prevention, Jinan, 250014 China; Freeman Spogli Institute, Stanford University, Stanford, CA 94305 USA; Weifang Medical College, Weifang, 261053 China

**Keywords:** Elderly, Empty-nest, Non-visiting, Non-hospitalization, China

## Abstract

**Background:**

Empty-nest elderly refers to those elderly with no children or whose children have already left home. Few studies have focused on healthcare service use among empty-nest seniors, and no studies have identified the prevalence and profiles of non-use of healthcare services among empty-nest elderly. The purpose of this study is to compare the prevalence of non-use of healthcare services between empty-nest and non-empty-nest elderly and identify risk factors for the non-use of healthcare services among empty-nest seniors.

**Methods:**

Four thousand four hundred sixty nine seniors (60 years and above) were draw from a cross-sectional study conducted in three urban districts and three rural counties of Shandong Province in China. Non-visiting within the past 2 weeks and non-hospitalization in previous year are used to measure non-use of healthcare services. Chi-square test is used to compare the prevalence of non-use between empty-nesters and non-empty-nesters. Multivariate logistic regression analysis is employed to identify the risk factors of non-use among empty-nest seniors.

**Results:**

Of 4469 respondents, 2667(59.7 %) are empty-nesters. Overall, 35.5 % of the participants had non-visiting and 34.5 % had non-hospitalization. Non-visiting rate among empty-nest elderly (37.7 %) is significantly higher than that among non-empty-nest ones (32.7 %) (*P* = 0.008). Non-hospitalization rate among empty-nesters (36.1 %) is slightly higher than that among non-empty-nesters (31.6 %) (*P* = 0.166). Financial difficulty is the leading cause for both non-visiting and non-hospitalization of the participants, and it exerts a larger negative effect on access to healthcare for empty-nest elderly than non-empty-nest ones. Both non-visiting and non-hospitalization among empty-nest seniors are independently associated with low-income households, health insurance status and non-communicable chronic diseases. The non-visiting rate is also found to be higher among the empty-nesters with lower education and those from rural areas.

**Conclusions:**

Our findings indicate that empty-nest seniors have higher non-use rate of healthcare services than non-empty-nest ones. Financial difficulty is the leading cause of non-use of health services. Healthcare policies should be developed or modified to make them more pro-poor and also pro-empty-nested.

## Background

China has the largest number of the older people in the world [1]. In 2013, 14.9 % of the Chinese population (202.4 million) were aged 60 and above [2]. Consistent with the global trend, China will step into a fastest aging period in its history [3, 4]. By 2050, the elderly is expected to represent 33 % of the total population [5, 6] . With the accelerating process of population aging trend, the absolute and relative numbers of empty-nest elderly families are both on the rise in China [7, 8]. Empty-nest elderly refers to those elderly with no children or whose children have already left home. These older people either live alone (empty-nest singles) or with a spouse (empty-nest couples) [9]. There were 100 million empty-nest older people in 2013, accounting for about 50 % of the total elderly population in China [10]. This proportion is projected to represent 90 % of the total aged population by 2030 [11].

To ensure equal access to health care according to needs, regardless of age, gender, ethnic background and capacity to pay, is an important goal for health service system worldwide [12, 13]. The aging process has raised concerns about equal access to health care for seniors, who are identified to have higher healthcare needs but often lack the financial capacity to pay [14]. Several studies have addressed this issue by examining associated factors with healthcare utilization among older people in China, including socio-demographic characteristics (e.g. age, gender), income, health insurance and healthcare needs [14–17]. Non-use of health care service (e.g. non-visiting, non-hospitalization) is another aspect of the health care access issue, which mainly results from limited availability or unavailability of health care services when they are needed, such as non-visiting and non-hospitalization [13]. It is one of the most important health care access issues we should address, as it may further result in more serious health problems and pose higher health burdens on individuals and households. Therefore, to assess non-use of health care service and its associated factors is of high priority. However, few studies have explored non-use of health care service and its associated factors among elderly in China.

The aging is accompanied by an increase in the prevalence of non-communicable chronic diseases (NCDs) and resultant disabilities [18, 19]. Some researchers indicate that empty-nest elderly are at higher risk of NCDs and other diseases than non-empty-nest ones. The empty-nesters also have less financial support from children [20, 21]. Thus, the empty-nest elderly may have higher prevalence of non-use of health care service (or poorer health care access) than non-empty-nest ones. To date, few studies have focused on the healthcare service use among empty-nest older people [21], no studies have identified the prevalence and profiles of non-use of health care services among empty-nest elderly. To remedy this situation, the present study aims to identify the non-use of healthcare among this special population. The overall goal of this study is to identify the prevalence of non-use of health care use and its associated factors among empty-nest older people. To do so, we have following specific objectives. First, we will compare the prevalence of non-use of health care services between empty-nest and non-empty-nest elderly. Second, we will identify the associated factors for non-use of health care services among the empty-nest elderly.

## Methods

### Study setting and study population

This study was conducted in Shandong province. It ranks the second in the number of total population in China. In 2012, the older people aged 60 and above accounted for 15 % of the total population (about 97 million) in Shandong. Among which, 50 % were empty-nesters [22].

**Fig. 1:**
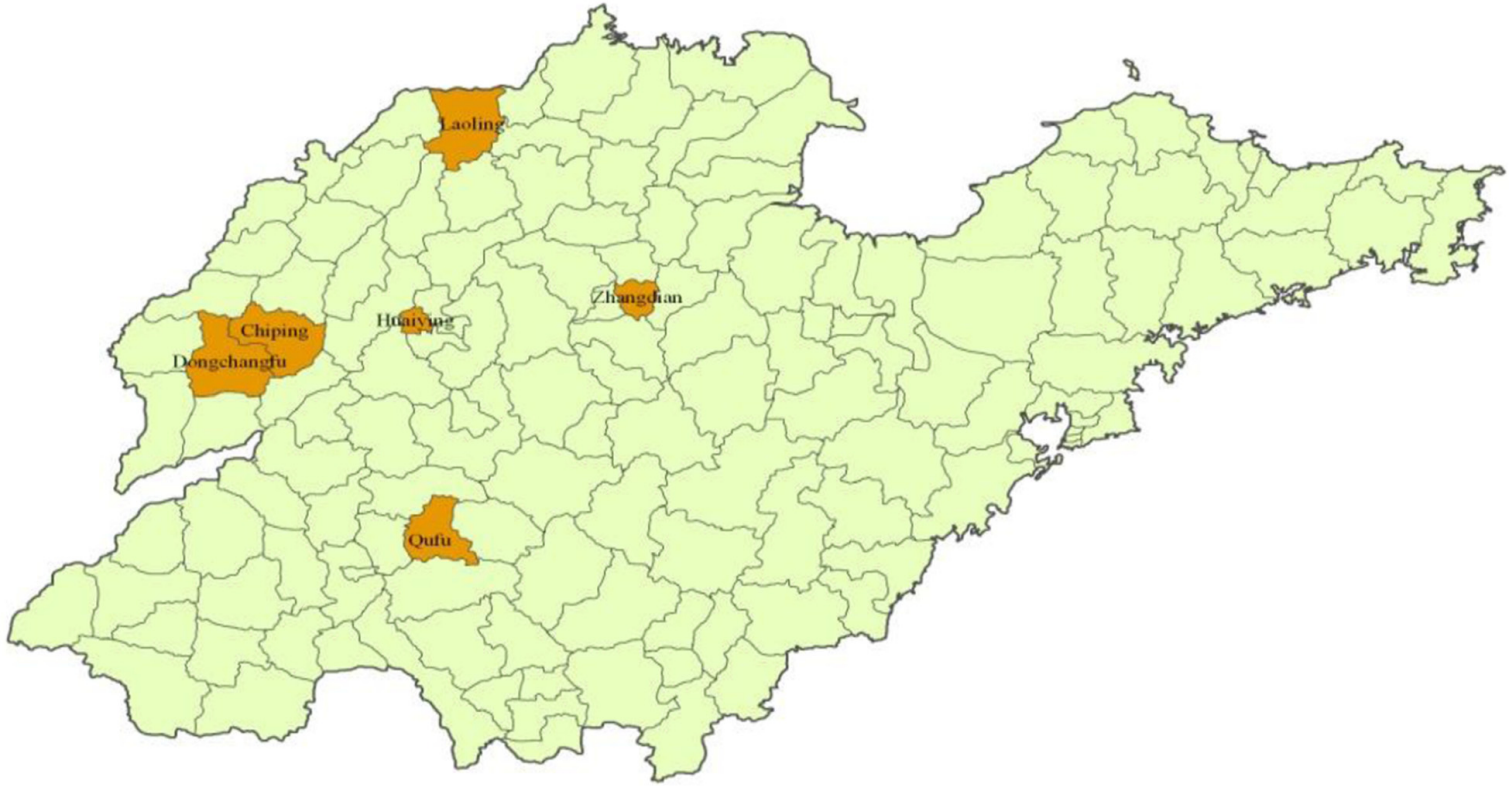
Location of the study sites in Shandong province, China

In this study, we used a 3-stage cluster sampling to select participants. First, we stratified all districts and counties in Shandong as three groups according to the GDP per capita (2011) respectively. Then we randomly selected one district and one county from each group. Three urban districts (Huaiyin, Dongchangfu and Zhangdian) and three rural counties (Qufu, Chiping and Leling) were selected as the study sites (see Fig. 1). Likewise, three sub-districts and three townships were then selected in each sampling district or county according the GDP per capita. Third, we randomly selected three communities and three villages from each selected sub-district or township. In total, 27 urban communities and 27 rural villages were selected. All of the elderly households of the sampling communities or villages were recruited into our survey. A total of 4469 older people with complete data are included in the analysis.

### Data collection

We collected the data from November 2011 to January 2012 by using a house-to house interview. All the elderly were interviewed face-to-face using a standard structured questionnaire by trained postgraduate students from Shandong University School of Public Health. To ensure quality, completed questionnaires were carefully checked by quality supervisors after the interview. The questionnaire included: household living arrangements, demographic information on individuals and households, self-rated health, health care needs, non-use of health care service and reasons.

### Variables

#### Dependent variables

Two measures of the non-use of health care service are used as dependent variables: one) “Non-visiting”, is defined as “not visit physicians despite being ill within the last 2 weeks; two) “Non-hospitalization”, is defined as “not using inpatient services despite being referred by doctors for hospital admission during the previous year.” In addition, the respondents were also asked about the reasons for non-visiting and non-hospitalization.

#### Independent variables

Based on the Andersen model [23], we categorize the independent variables into three types: predisposing, enabling and need variables. *a.) Predisposing variables.* In the present paper, the variables include age (60−, 70−, 80+), gender (male, female), education (primary and below, junior, high and above), and marital stauts (married or others). *b.) Enabling variables.* We classify residence (urban, rural), empty-nester living arrangements (empty-nest single or living alone, empty-nest couple or living with a spouse), low-income households (In Chinese, we call it “Dibaohu”) which was identified by local governments and subsidized by local bureau of civil affairs, and health insurance (Medical insurance for urban employees scheme (MIUE), Medical insurance for urban residents scheme(MIUR),New cooperative medical scheme (NCMS), Others, such as commercial insurance). *c.) Need variables.* Self-rated health (good, moderate, bad)*,* illness in the past 2 weeks and NCDs in the past 6 months are used as measures of needs.

### Data analysis

The data was double entered and checked using EPI Data 6.04. The statistical package SPSS 13.0 was used to analyze the data. Chi-square test was used to compare non-use of health care between empty-nest and non-empty-nest elderly. Reasons for non-use of health care were presented as percentages. Preliminary analyses were performed firstly using univariate logistic regression to check which factors were associated with non-use of health care. Multivariate logistic regression was then employed to assess the explanatory variables for non-visiting and non-hospitalization. Statistical significance was set at the 5 % level.

### Ethical consideration

The Ethical Committee of Shandong University School of Public Health approved the study protocol. The investigation was conducted after the informed consents of all participants were obtained.

## Results

Of respondents, 2667(59.7 %) are empty-nesters and 1802(40.3 %) are non-empty-nesters (see Table 1). Females are more than males both among empty-nesters and non-empty-nesters. Non-empty-nesters with the age from 60 to 69 account for 65.7 %, which is higher than that among empty-nesters (58.8 %). The proportion of married elderly among empty-nesters (82.9 %) is higher than that among non-empty-nesters (72.2 %). Urban elderly account for 52.6 % and 50.0 % among empty-nesters and non-empty-nesters respectively. The overall proportion of the elderly from low-income households is 7.4 %, and the two groups have no significant difference. Among the empty-nesters, empty-nest singles account for 17.1 %, while empty-nest couples account for 82.9 %. 55.4 % of the non-empty-nesters report good perceived health, which is significantly higher than that of non-empty-nesters (47.9 %). Prevalence rate of illness within 2 weeks before the survey and prevalence rate of chronic disease among empty-nesters and non-empty-nesters are 68.6 %, 67.8 % and 59.2 %, 62.2 % respectively, the differences in the above two indicators are statistically significant between the two groups.

**Table 1:** Characteristics of elderly by living arrangements, China (2012)

Characteristics	Total		Empty-nest		Non-empty-nest		*P*
	n	%	n	%	n	%	
Observations	4469	100.0	2667	59.7	1802	40.3	
Predisposing factors							
*Gender*							0.000
Male	2078	46.5	1286	48.2	792	44.0	
Female	2391	53.5	1381	51.8	1010	56.0	
*Age*							0.000
60−	2629	58.8	1445	54.2	1184	65.7	
70−	1429	32.0	968	36.3	461	25.6	
80+	411	9.2	254	9.5	157	8.7	
*Marriage status*							0.000
Married	3511	78.6	2210	82.9	1301	72.2	
Others	958	21.4	457	17.1	501	27.8	
*Education*							0.100
Primary or below	3145	70.4	1849	69.3	1296	71.9	
Junior	713	16.0	431	16.2	282	15.6	
High or above	611	13.7	387	14.5	224	12.4	
Enabling factors							
*Residence*							0.083
Urban	2305	51.6	1404	52.6	901	50.0	
Rural	2164	48.4	1263	47.4	901	50.0	
*Living arrangements*					NA^b^		
Empty-nest single			457	17.1			
Empty-nest couple			2210	82.9			
*Low income household* ^a^							0.385
Yes	331	7.4	205	7.7	126	7.0	
No	4138	92.6	2462	92.3	1676	93.0	
*Insurance* ^c^							0.000
None	193	4.3	117	4.4	76	4.2	
MIUE	1168	26.1	776	29.1	392	21.8	
MIUR	545	12.2	327	12.3	218	12.1	
NCMS	2518	56.3	1417	53.1	1101	61.1	
Others	45	1.0	30	1.1	15	0.8	
Need factors							
*Self-rated health*							0.000
Good	2277	51.0	1278	47.9	999	55.4	
Moderate	1497	33.5	939	35.2	558	31.0	
Bad	695	15.6	450	16.9	245	13.6	
*Illness in the past 2 week*							0.000
Yes	2896	64.8	1830	68.6	1066	59.2	
No	1573	35.2	837	31.4	736	40.8	
*NCD in the past 6 months* ^d^							0.000
Yes	2930	65.6	1809	67.8	1121	62.2	
No	1539	34.4	858	32.2	681	37.8	

^a^Low income household: In Chinese, we call it “Dibaohu”, which is identified as a low-income household and subsidized by local bureau of civil affairs

^b^NA: not applicable

^c^MIUE: Medical insurance for urban employees scheme; MIUR: Medical insurance for urban residents scheme; NCMS: New cooperative medical scheme

^d^NCD: Non-communicable chronic disease

Table 2 shows the non-use of health care services among the elderly. Overall, prevalence of non-use but ought-to-use physician visit within the last 2 weeks is 35.5 %, and the prevalence of non-use of in-patient service is 34.5 %. When comparing the non-use of healthcare between the two subgroups, we find that non-visiting rate among empty-nest elderly (37.7 %) is significantly higher than that among non-empty-nest elderly (32.7 %) (*P* = 0.008). The result also shows that non-hospitalization rate among empty-nesters (36.1 %) is a little higher than that among non-empty-nesters (31.6 %), but the difference has no statistical significance (*P* = 0.166).

**Table 2:** Comparison of non-use of health care between empty-nest and non-empty-nest elderly, China (2012)

Non-use of health care	Total	Empty-nest (%)	Non-empty-nest(%)	*P*-value
*Outpatient service non- use*				0.008
Physician visits	1858(64.2)	1141(62.3)	717(67.3)	
Non-visiting	1038(35.8)	689(37.7)	349(32.7)	
*Inpatient service non-use*				0.166
Admission	618(65.5)	391(63.9)	227(68.4)	
Non-hospitalization	326(34.5)	221(36.1)	105(31.6)	

Among the participants, 689 empty-nesters and 349 non-empty-nesters reported they did not visit physicians when they had illness within the last 2 weeks, 221 empty-nesters and 105 non-empty-nesters reported they didn’t use in-patient service despite being recommended by physicians for admission. Table 3 presents the reasons for non-use of health care services. The leading cause for non-visiting among empty-nester is “financial difficulties”(46.4 %), followed by “illness not serious” (31.3 %). As for non-empty-nesters, 43.3 % reports “illness not serious” for non-visiting, followed by financial difficulties (33.3 %). The difference of the reasons for non-visiting between empty-nesters and non-empty-nesters is statistically significant(*χ*^2^ =23.41, *P* = 0.000). Likewise, the leading reason for non-hospitalization is financial difficulties for both of the two groups (57.9 % among empty-nesters and 47.6 % among non-empty-nesters), followed by “illness not serious” (21.3 % among empty-nesters and 27.6 % among non-empty-nesters). Without effective treatment also limited their physician visiting service and in-patient service to some degree. But the difference of the reasons for non-hospitalization between the two groups has no statistical significance (*χ*^2^ =4.23, *P* = 0.518).

**Table 3:** Reasons for non-use of health service among elderly in Shandong, China (%) (2012)

Reasons		Non-visiting			Non-hospitalization	
	Total (%)	Empty-nesters (%)	Non-empty-Nesters (%)	Total (%)	Empty-nesters (%)	Non-empty-nesters (%)
N	1038	689	349	326	221	105
Financial difficulties	436(42.0)	320(46.4)	116(33.3)	178(54.6)	128(57.9)	50(47.6)
Illness not serious	366(35.3)	215(31.3)	151(43.3)	76(23.3)	47(21.3)	29(27.6)
Without effective medical treatment	59(5.7)	44(6.4)	15(4.3)	29(8.9)	18(8.1)	11(10.5)
Poor transportation	17(1.6)	9(1.3)	8(2.3)		--	--
Inconvenient	14(1.3)	8(1.2)	6(1.7)		--	--
No enough time	--	--	--	8(2.5)	4(1.8)	4(3.8)
No beds	--	--	--	3(0.9)	2(0.9)	1(1.0)
Others	145(14.0)	92(13.4)	53(15.2)	32(9.8)	22(10.0)	10(9.5)
		*χ* ^2^ =23.41, *P* = 0.000			*χ* ^2^ =4.23, *P* = 0.518	

We compare the non-visiting rate across different subgroups of empty-nest elderly using univariate analysis (Table 4). The output shows that those empty-nesters who are married (*P* = 0.039), who have educational level of high and above (*P* = 0.000), who live in urban areas (*P* = 0.000), who are covered by MIUE (*P* = 0.005) and MIUR (*P* = 0.025) are less likely to experience non-visiting. Those who are from low-income households, who have no NCDs within the past six months are more likely to experience non-visiting. Multi-logistic regression analysis is used to identify the determinants linked with the non-visiting. Empty-nesters who have high school and above education, who live in urban areas, who are covered by NCMS are less likely to have non-use of physician visit. Empty-nesters who are from low-income households and who have no NCDs in the previous six months tend to experience non-visiting.

**Table 4:** Factors associated with non-visiting among empty-nest elderly in Shandong, China (2012)

Variable	*Univariate model*				*Multivariate model*		
	*Non-user (%)*	*P-value*	*OR*_*c*_^*a*^	*OR*_*c *_ *95 % CI*	*P-value*	*OR*_*a*_^*b*^	*OR*_*a*_ *95 % CI*
Predisposing factors							
*Gender*					NA^c^		
Male	304(35.7)		1.0				
Female	385(39.3)	0.113	1.17	0.96–1.41			
*Age*					NA		
60−	349(37.1)		1.0				
70−	272(39.0)	0.438	1.08	0.89–1.33			
80+	68(35.6)	0.698	0.94	0.68–1.30			
*Marriage status*							
Married	540(36.5)		1.0			1.0	
Others	149(42.5)	0.039	1.28	1.01–1.63	0.217	1.17	0.91–1.49
*Education*							
Primary and below	519(40.6)		1.0			1.0	
Junior	99(35.0)	0.082	0.79	0.60–1.03	0.469	0.90	0.68–1.20
High or above	71(26.5)	0.000	0.53	0.39–0.71	0.007	0.64	0.46–0.88
Enabling factors							
*Residence*							
Urban	322(31.9)		1.0			1.0	
Rural	367(44.7)	0.000	1.73	1.43–2.09	0.002	1.69	1.21–2.35
*Living arrangements*							
Empty-nest singles	149(42.5)		1.0			1.0	
Empty-nest couples	540(36.5)	0.039	0.78	0.62–0.99	0.217	1.17	0.91–1.49
*Low-income household* ^*d*^							
No	613(36.4)		1.0			1.0	
Yes	76(51.4)	0.000	1.84	1.31–2.58	0.008	1.60	1.13–2.56
*Insurance* ^e^							
None	37(46.8)		1.0				
MIUE	172(30.8)	0.005	0.51	0.31–0.81	0.059	0.62	0.38–1.02
MIUR	81(32.8)	0.025	0.55	0.33–0.93	0.056	0.60	0.35–1.01
NCMS	389(42.1)	0.414	0.83	0.52–1.31	0.022	0.54	0.32–0.92
Others	10(47.6)	0.949	1.03	0.39–2.71	0.842	1.11	0.41–2.96
Need factors							
*Self-rated health*					NA		
Good	241(36.5)		1.0				
Moderate	280(36.8)	0.899	1.01	0.82–1.26			
Bad	168(41.0)	0.145	1.21	0.94–1.55			
*NCD in the past 6 months* ^f^							
Yes	651(36.9)		1.0			1.0	
No	38(57.6)	0.001	2.32	1.41–3.82	0.003	2.16	1.30–3.58

^a^OR_c_: crude odds ratio; ^b^OR_a_: adjusted odds ratio

^C^NA: not applicable

^d^Low income household: In Chinese, we call it “Dibaohu”, which is identified as a low-income household and subsidized by local bureau of civil affairs

^e^MIUE: Medical insurance for urban employees scheme; MIUR: Medical insurance for urban residents scheme; NCMS: New cooperative medical scheme

^f^NCD: Non-communicable chronic disease

Table 5 shows the influencing factors associated with non-hospitalization. The univariate analysis indicates that empty-nesters who are covered by MIUE (*P* = 0.000), MIUR (*P* = 0.000) and NCMS (*P* = 0.000) are less likely to experience non-hospitalization. Those who are from low-income households (*P* = 0.000), who have no NCDs within the past six months (*P* = 0.039) are more likely to experience non-hospitalization. Multi-logistic regression analysis identifies that the above three factors are the determinants linked with the non-hospitalization.

**Table 5:** Factors associated with non-hospitalization among empty-nest elderly in Shandong, China (2012)

Variable	*Univariate model*				*Multivariate model*		
	*Non-user (%)*	*P-value*	*OR*_*c*_^*a*^	*OR*_*c*_ *95 % CI*	*P-value*	*OR*_*a*_^*b*^	*OR*_*a*_ *95 % CI*
Predisposing factors							
*Gender*					NA ^c^		
Male	107(37.0)		1.0				
Female	114(35.3)	0.656	0.93	0.67–1.29			
*Age*					NA		
60−	95(35.1)		1.0				
70−	94(36.7)	0.691	1.08	0.75–1.54			
80+	32(37.6)	0.664	1.12	0.68–1.85			
*Marriage status*					NA		
Married	173(34.7)		1.0				
Others	48(42.1)	0.141	1.37	0.90–2.07			
*Education*					NA		
Primary and below	138(35.4)		1.0				
Junior	37(38.5)	0.564	1.15	0.72–1.82			
High or above	46(36.5)	0.819	1.05	0.69–1.60			
Enabling factors							
*Residence*					NA		
Urban	142(35.9)		1.0				
Rural	79(36.6)	0.860	1.03	0.73–1.46			
*Living arrangements*					NA		
Empty-nest singles	48(42.1)		1.0				
Empty-nest couples	173(34.7)	0.141	0.73	0.48–1.11			
*Low-income household* ^d^							
No	190(33.8)		1.0			1.0	
Yes	31(62.0)	0.000	3.19	1.76–5.80	0.005	3.55	1.91–6.59
*Insurance* ^e^							
None	29(76.3)		1.0			1.0	
MIUE	79(33.9)	0.000	0.16	0.07–0.35	0.000	0.17	0.07–0.37
MIUR	29(34.5)	0.000	0.16	0.07–0.39	0.000	0.16	0.07–0.39
NCMS	80(32.1)	0.000	0.15	0.07–0.33	0.000	0.13	0.06–0.30
Others	4(50.0)	0.145	0.31	0.06–1.50	0.161	0.32	0.07–1.57
Need factors							
*Self-rated health*					NA		
Good	55(31.8)		1.0				
Moderate	97(38.6)	0.149	1.35	0.90–2.03			
Bad	69(36.7)	0.327	1.24	0.80–1.92			
*Illness in the past 2 weeks*					NA		
Yes	200(35.6)		1.0				
No	21(42.0)	0.367	1.31	0.73–2.36			
*NCD in the past 6 months* ^f^							
Yes	203(35.1)		1.0			1.0	
No	18(52.9)	0.039	2.08	1.04–4.16	0.027	2.24	1.10–4.59

^a^OR_c_: crude odds ratio; ^b^OR_a_: adjusted odds ratio; ^C^NA: not applicable

^d^Low income household: In Chinese, we call it “Dibaohu”, which is identified as a low-income household and subsidized by local bureau of civil affairs

^e^MIUE: Medical insurance for urban employees scheme; MIUR: Medical insurance for urban residents scheme; NCMS: New cooperative medical scheme

^f^NCD: Non-communicable chronic disease

## Discussion

One of the main features of China’s population aging is the transition of elderly household patterns. To live with children is a typical Chinese traditional family pattern for older people [21]. However, with the implementation of 1-child policy, the imbalance of economic development, and the acceleration of urbanization, the number of empty-nest families is on a rapid rise in the past decades. Our findings show that 59.7 % of the elderly in our sample are empty-nesters. This proportion is higher than the reported proportion of 49 % in 2012 in the same province [22]. According to this figure, we estimate there are about 10 million of elderly living without children in Shandong province. Among which, about 1.7 million are empty-nest singles.

Consistent with previous reports, our study finds that the empty-nest elderly have poorer self-rated health, higher prevalence of 2-week illness and NCDs, which indicates that the empty-nesters have poorer health status (or higher healthcare needs) than non-empty-nest elderly [20, 24]. The departure of the children, the most important source of the love feelings and social support for the elderly, will probably increase the loneliness and also negatively affect the quality of life of the empty-nest elderly. Such feelings of loneliness and poor quality of life will push many older people into a so-called empty-nest syndrome [7]. Many researchers have pointed out that the empty-nest syndrome could result in endocrine disorders, immune dysfunction, and further cause various diseases (e.g. cardiovascular disease, cancer) [7, 25]. As such, empty-nest elderly may have higher health care need than non-empty-nest ones. Organizing senior associations might help improve the empty-nesters’ mental health.

Ideally, health service system is established to ensure equal access to health care primarily based on health needs. If this is true, it could be that empty-nesters should experience at least as equal prevalence of non-use of health care as non-empty-nesters. However, our findings reveal that empty-nest older people receive statistically higher rate of non-visiting and slightly higher prevalence of non-hospitalization than non-empty-nest ones, which indicates an inequality of health service access between the two subgroups of the seniors. This finding suggests that it is very important to explore the reasons for the higher prevalence of non-use of health care among empty-nesters and develop targeting interventions to enhance accessibility for such vulnerable population.

Some previous studies indicated that financial difficulty is the key barrier to access of health care service [15, 20, 26]. In this study, the analysis of the self-reported reasons for non-use of health services among the elderly also shows that financial difficulty is the leading cause for giving up seeking out-patient and in-patient services when needed. When comparing with non-empty-nest seniors, we found that financial difficulty exerts a statistically larger negative effect on access to out-patient service and also a larger negative effect on access to in-patient health service (even though the difference is not statistically significant) among empty-nest ones, which may be associated with their lower income and less economic support from children than the non-empty-nest ones. Surprisingly, our findings show that over 46 % of the empty-nest seniors attribute their non-visiting to financial difficulty. One possible explanation for this phenomenon is that high prevalence of NCDs push the empty-nesters into a status of using health service repeatedly and pose a high economic burden on them. Another explanation might be the shortcomings of the health insurance policy design for the low reimbursement rate or ceiling of the out-patient services. Furthermore, we also observe that nearly 58 % of the empty-nesters report the reason of financial difficulty for non-hospitalization. These findings indicate a need for the government to redesign health insurance policies to improve the accessibility to healthcare by extending the out-patient benefit package and increasing reimbursement rate for in-patient service for empty-nest elderly.

An interesting finding in this study is that the empty-nesters with NCDs experience statistically lower non-use in both physician visits and hospitalization than those without NCDs, which is inconsistent with other studies conducted in general older people [14]. There are two possible explanations for such finding. First, as a population lacking of social support, empty-nesters will consider more about the serious consequences (e.g. disability) of non-use of NCDs-related healthcare service when needed. Second, NCDs are not easily curable and need more frequent physician visits. As a result, the empty-nesters being chronically ill will well understand the symptoms and severity of NCDs and know the importance of timely visit. This finding needs to be further studied to verify among the empty-nesters.

Similar with other studies, our findings reveal that empty-nesters from low-income family tend to experience non-visiting and non-hospitalization when adjusting for need factors and predisposing factors [14, 21]. We also find that health insurance schemes (e.g. NCMS) can significantly reduce the non-use of physician visits and hospitalization among the empty-nest seniors. The findings should therefore give an impetus to develop pro-poor and pro-empty-nested health insurance policies. A special insurance supplement to existing schemes (e.g. NCMS) or a combination of health insurance and medical assistance policy targeting poor empty-nesters may be effective to reduce non-use of healthcare when needed. It is also noted in the present study that empty-nesters with lower education and those from rural areas are more likely to have non-visiting, which indicates inequality of access to healthcare service among such subgroups.

The present study involves certain limitations. First, we use the period of 2 weeks to control recall bias when measuring non-use of physician visits. However, the potential issue of seasonal variation, particularly for those acute diseases, is unavoidable. Second, information including perceived health status, healthcare use and reasons for non-use of healthcare were self-reported, leading to the possibility of subjective bias. Third, a cross-sectional design is employed in this study, therefore, the relationship between identified factors and non-use of healthcare use can not be interpreted as cause and effect.

## Conclusion

This study shows that empty-nest seniors have higher healthcare needs than non-empty-nest ones. We observe an inequality of healthcare access between empty-nesters and non-empty-nesters. Financial difficulty is the leading cause for the non-use of healthcare service among the two sub-groups of seniors and such cause exerts a larger negative effect on access to healthcare among empty-nesters. The findings indicate some at-risk subgroups of empty-nesters for non-use of physician visiting and hospitalization, such as those from low-income family, the uninsured, and those without NCDs. A comprehensive pro-poor policy of a supplement to existing health insurance schemes or a combination of health insurance and medical assistance is necessary to increase healthcare use when needed among empty-nesters.
